# Is the Rivermead Post-Concussion Symptoms Questionnaire a Reliable and Valid Measure to Assess Long-Term Symptoms in Traumatic Brain Injury and Orthopedic Injury Patients? A Novel Investigation Using Rasch Analysis

**DOI:** 10.1089/neur.2020.0017

**Published:** 2020-08-11

**Authors:** Shivanthi Balalla, Chris Krägeloh, Oleg Medvedev, Richard Siegert

**Affiliations:** ^1^ Department of Psychology and Neuroscience, Auckland University of Technology, Northcote, Auckland, New Zealand.; ^2^ School of Psychology, University of Waikato, Hamilton, New Zealand.

**Keywords:** brain injuries, orthopedic injuries, post-concussion syndrome, psychometrics, Rasch analysis

## Abstract

Persistent post-concussion syndrome (PCS) symptoms are known to last years after traumatic brain injury (TBI), and similar symptoms are increasingly being documented among those who have not experienced a TBI. There remains however, a dearth of empirical evidence on the structural composition of symptoms beyond the post-acute symptom phase after TBI, and little is known about the potential use of PCS symptom scales to measure PCS-like symptoms in non-TBI individuals. Our objective was therefore to examine the psychometric performance and dimensionality of the Rivermead Post-Concussion Symptoms Questionnaire (RPQ) as a measure of long-term PCS symptoms among a TBI and non-TBI sample. A case-control sample of 223 patients with injury, consisting of age- and sex-matched TBI participants (*n* = 109) and orthopedic participants (*n* = 114) were recruited from a regional trauma registry in New Zealand (NZ), and assessed at mean 2.5 years post-injury. Results from the Rasch analysis showed that the RPQ achieved fit to the Rasch model, demonstrating very good reliability (Person Separation Index [PSI] = 0.87), thereby indicating that the measure can be used reliably for individual and group assessment of symptoms among both TBI and orthopedic patients. In this study we demonstrated evidence of a unidimensional construct of PCS symptoms in both groups, which helps alleviate previous uncertainty about factor structure, and permits the calculation of a total RPQ score. Conversion of ordinal to interval total scores presented within are recommended for clinicians and researchers, to improve instrument precision, and to facilitate the interpretation of change scores and use of parametric methods in data analysis.

## Introduction

Traumatic brain injury (TBI) has myriad consequences that can have lasting effects on cognition, physical and psychological functioning, return to employment, social reintegration, and quality of life.^1–3^ Among the most commonly persisting difficulties associated with TBI is the onset of post-concussion syndrome (PCS) symptoms, which are a constellation of neurological and neuropsychological symptoms including headache, dizziness, irritability, anxiety, depression, fatigue, and difficulties with memory and concentration.^4^ Symptoms of PCS are particularly prevalent among those who have experienced mild TBI,^5^ and are known to last from hours after trauma up to 10 years after injury.^6,7^ Factors such as cognitive and somatic symptoms immediately after injury, pre-existing depression, past history of TBI, and sleep quality have been found to increase the chronicity of symptoms.^8^

The Rivermead Post-Concussion Symptoms Questionnaire (RPQ)^9^ is one of the most widely used scales to assess PCS symptoms following TBI, and has demonstrated good internal consistency, test-retest, and inter-rater reliability.^9–11^ Previous research utilizing both classical approaches of factor analysis and more modern psychometric techniques such as Rasch analysis has consistently pointed to multi-dimensionality existing within the scale, especially when used with patients in the early recovery period.^11–13^ Variable factor structures have been proposed comprising two, three, or four dimensions underpinning PCS symptoms; however, there is a lack of consensus as to the most consistent structure.^14^ Further fueling the ongoing debate is the non-specificity of PCS symptoms as a phenomenon solely attributable to the experience of a TBI, given that symptoms arise in various groups including those with chronic pain,^15^ psychological disorders,^16^ orthopedic injuries,^17^ and even within the healthy population.^8^ Despite the burgeoning evidence across such diverse samples, the psychometric utility of these PCS symptom scales has not yet been examined in non-TBI populations.

The application of modern psychometric approaches such as Rasch analysis for scale validation offers several advantages over factor analysis. The Rasch measurement model accounts for the probability of a person endorsing a Likert-scale item at a particular value as a function of two important parameters—the difficulty of an item and the amount of a trait held by a person, or the so-called *person ability*.^18^ These two parameters are represented on the same logarithmic interval scale, which is a central tenet of the Rasch model. An added advantage of Rasch analysis is that it offers detailed information about the performance of individual items, including to what extent an item functions consistently by a demographic feature such as by age group, sex, or injury type. An important feature of Rasch analysis is that once a measure has been shown to meet the requirements of the Rasch model, ordinal scales can be transformed to an interval-level scale that not only ensures measurements are more precise, but also permits the use of more robust parametric data analyses without violating fundamental assumptions. As this transformation adjusts for differences in item difficulty, scores on the Rasch model interval scale therefore represent a more accurate reflection of a person's trait level.

Rasch analysis can present novel ways to resolve current ambiguities around dimensionality of the RPQ in the TBI population, which may be underpinned by issues such as *local response dependency* or *local trait dependency*. Past attempts to resolve discrepancies around factor structure have been conducted with the application of Rasch methods, but as of yet they have not resulted in a consistent solution.^11,12^ Previous examinations of the RPQ have also not extended beyond the first year after TBI, with many focussing only on the acute symptom phase, namely 3–6 months post-TBI. Symptoms are known to persist many years after injury,^6,7,13^ and currently there remains a dearth of evidence regarding the structural composition of symptoms in the mid-to-late recovery period after TBI.

The present Rasch investigation aimed to elucidate on the obscurities regarding the dimensionality of the RPQ by utilizing a subtest approach to control for method effects, to evaluate long-term post-concussion symptoms that are present at approximately 2.5 years after TBI. As validation of PCS symptom scales in non-TBI populations does not currently exist, this study also aimed to examine whether the RPQ can be reliably administered as a measure of PCS-like symptoms, in a control injury sample consisting of participants with orthopedic injuries. In line with best practice guidelines for Rasch analyses^19^ we have presented conversion tables for raw total-ordinal to total-interval scores to increase instrument precision. These transformations enable clinicians and researchers to accurately use summary scores to aid both group and individual comparisons.

## Methods

### Participants

Patients diagnosed with a TBI or an orthopedic injury and discharged alive between 2012 and 2016 (*n* = 1090), were recruited for initial contact from a regional hospital registry in the Waikato region of New Zealand (NZ). Injuries were defined by relevant Abbreviated Injury Scale 2005–08 codes.^20^ As per World Health Organization guidelines^21^ a TBI diagnosis was recorded in the registry where there was evidence of a cerebellum injury, hematoma, contusion, diffuse axonal injury, brain swelling, loss of consciousness, alteration of mental state, or presence of post-traumatic amnesia. The TBI group consisted of both isolated TBI and multiple-injury TBI cases (TBI and extracranial injuries). Orthopedic injuries included joint injuries relating to fractures, dislocations, or sprains to the pelvic region, and upper and lower extremities. Orthopedic injuries resulting from “insufficiency” or stress fractures, pre-existing medical conditions (e.g., epilepsy, Parkinson's disease), or without evidence of external force were excluded. Participants who were unable to give consent due to language barriers (i.e., non-English speaking) or who had cognitive or hearing impairments were excluded, as well as those who had likely experienced significant psychological trauma due to the nature of their injuries (e.g., crushes and amputations).

Clinical injury details including Glasgow Coma Scale (GCS) score and Injury Severity Score (ISS) were obtained from registry data. Follow-up interviews were conducted between 6 months and 6 years post-injury and administered via telephone interview by the primary investigator. Informed consent was obtained through audio-tape recording. Final response rates of 38% and 39%, yielded a total of 109 TBI and 114 orthopedic cases, respectively, with participants being matched by age (5-year bands) and sex. There were no significant differences between respondents and non-respondents except for ethnic group and discharge destination. Ethical approval was granted by the institutional, hospital, and national ethics committees.

### Outcome measures

The RPQ is a 16-item self-report questionnaire that assesses the severity of 16 different PCS symptoms that typically follow TBI. Participants were asked to rate their symptoms before and after their injury and also to rate the severity of their symptoms in the last 24 h. Items follow a 5-point ordinal rating system where 0 = never experienced at all, 1 = no more of a problem, 2 = a mild problem, 3 = a moderate problem, and 4 = a severe problem. Thus total scores using the sum of all items can theoretically range from 0 to 64,^12^ although due to reported differences in dimensionality there is no gold standard on the scale's scoring structure.^22^ The Cumulative Illness Rating Scale (CIRS)^23^ to assess comorbidity, and the World Health Organization Quality of Life Brief Version (WHOQoL-BREF) tool to assess health-related quality of life^2^ were also administered at the time of interview.

### Sample descriptives and data analysis

Descriptive statistics were calculated using IBM SPSS software version 25. Except for GCS scores, missing data were less than 1% and at random order. The sample size met the requirements to estimate person measures to ±0.5 logits within 99% confidence intervals for Rasch analyses,^24^ and was powered at 0.80 beta to detect moderate effect sizes between *d* = 0.30–0.40, at 0.05 alpha. Table 1 presents the sample demographics and clinical characteristics. Mean injury time between TBI and orthopedic groups was 2.5 and 2.7 years respectively (*p* = 0.289). Both injury groups were classified as minor trauma (ISS <16) but participants with TBI had marginally higher injury severity than orthopedic patients, ISS 11.0 and 4.0, respectively (*p* < 0.001), as well as longer hospital stays (*p* < 0.05). For the TBI sample, median GCS of 14.0 (of the 66% of GCS scores that were available) indicated a predominance of mild TBI.

**Table 1.** Sample Characteristics by Injury Group

Characteristic		TBI (n = 109)	Orthopedic (n = 114)	P-value
Mean age, years (SD)		48.8 (19.7)	48.0 (19.7)	0.905^a^
Median ISS		11.0	4.0	0.000^*,b^
Median LOS		6.0	4.0	0.036^*,b^
Mean time since injury, years (SD)		2.5 (0.13)	2.7 (0.04)	0.289^a^
Sex	Male	63.3	64.9	0.802^c^
Ethnic group %	NZ European	60.6	72.8	0.052^c^
	Other	39.4	27.2	
Education %	Primary/High school	54.1	43.9	0.125^c^
	Polytechnic/University	45.9	56.1	
Marital status %	Single	46.3	40.7	0.402^c^
	Living as married	53.7	59.3	

^*^ Significant at *p* < 0.05.

^a^ *t* test; ^b^Mann-Whitney U; ^c^χ^2^ test.

“Other” ethnic group consists of Māori, Pacific, Asian, and other ethnic minority groups; “Single” status refers to divorced, separated, widowed, and never married; “Living as married” refers to de facto and married.

ISS, Injury Severity Score; LOS, length of hospital stay in days; NZ, New Zealand; SD, standard deviation; TBI, traumatic brain injury.

For Rasch analyses, similar procedures employed by other Rasch studies^2,25^ were followed using the Rasch Unidimensional Measurement Model (RUMM) 2030 software,^26^ including the assessment of item-level and overall fit to the Rasch model, scale reliability, unidimensionality, scale targeting, threshold ordering, response dependency, and differential item functioning (DIF). A likelihood ratio test indicated the appropriate use of the polytomous Partial Credit Model^27^ over the Rasch Rating Scale Model^28^ (χ^2^[44] = 150.60, *p* < 0.001). Super-items or subtests were also created by merging related items to correct for method effects and to address deviations of the Rasch model. Further details of each procedure are presented in Table 2.

**Table 2.** Description of Procedures Undertaken for Rasch Analysis

Concept examined	Description of procedure
Overall Rasch model fit	Where data fit the Rasch model, chi-square (χ^2^) estimate should indicate that item-trait interaction is not significant (Bonferroni adjusted *p* > 0.05), and means and standard deviations for overall item and person fit residuals are close to 1.00 and 0.00, respectively. A significant χ^2^ reflects the presence of *local trait dependency*, indicating that the hierarchical ordering of items (item difficulty) varies across the trait or construct.^17^
	Fit residuals for individual items should also fall in the range of -2.50 to +2.50 to demonstrate fit to the Rasch model.^40^
Unidimensionality	Presence of unidimensionality is assessed by a principal component analysis of residuals where no further remaining associations among residuals are detected once the latent trait or Rasch component has been extracted. This is indicated by an independent *t* test where the percentage of significant *t* tests beyond +1.96 confidence intervals is <5%, and/or the lower bound confidence interval of significant tests overlaps the 5% mark.^41^
Reliability	Scale reliability is denoted by the Person Separation Index (PSI), which is equivalent to Cronbach's alpha. Values >0.70 permit group comparisons, and values >0.85 allow for individual assessment.^42^
Targeting of persons and items	For ideal targeting of a measure to person ability, the mean location should be centred around 0.00. Positive values indicate that the sample is located at a higher level of the construct, compared with the average of the scale, and the converse would be true for negative values.
Differential item functioning	Differential item functioning (DIF) refers to the effect that a particular item does not perform consistently across demographic features. In this study, DIF was assessed by age, sex, marital status (single/living as married), ethnic group (NZ European vs. Other), education (Primary/High school vs. Polytechnic/University), injury group (TBI vs. orthopedic), and time since injury (0-2.5 years vs. 2.5-6 years). Further, using a post hoc sign test of significance, the distinction was made between real DIF (or significant DIF) and artificial DIF, where the latter is said to occur spuriously as an artifact of the method for detecting DIF.^43^
Ordering of response thresholds	Response thresholds were examined visually using category probability curves. The presence of significantly disordered response thresholds would indicate that item response categories are problematic and do not work as expected.
Local response dependency	Presence of local response dependency (indicating item responses influencing one another) can be examined by looking at values that exceed +0.20 of mean residual correlations.^44^
Merging of related items: “super-items”	Violations of Rasch model assumptions including the presence of DIF, local dependency, and threshold disordering were addressed with the pairing of related items (based on their residual correlations) to create subtests or “super-items.” Super-items attenuate measurement error that exists within individual items.^45^

NZ, New Zealand; TBI, traumatic brain injury.

Where unidimensionality was met, a total score was derived using the sum of all 16 items.^12^ In line with guidelines for Rasch studies,^19^ presented in Supplementary Table S1 are conversions of raw ordinal to interval total scores (adjusted for both person ability/trait level and item difficulty), to improve accuracy where total scores are used.

## Results

Item-level fit statistics are presented in Table 3 alongside percentage of symptom endorsement by injury group. Distribution patterns of symptom endorsement were similar in both groups, with most participants reporting that each symptom was either not experienced post-injury or was not a current problem. In the total sample, only 1.8% of participants with TBI and 14.5% of orthopedic participants had not experienced any symptoms after injuries (χ^2^_Yates_[1] = 9.59, *p* = 0.002). In comparison, 67.9% TBI and 37.7% orthopedic participants endorsed experiencing at least one current PCS symptom (χ^2^_Yates_[1] = 20.34, *p* < 0.001), out of which the majority (approximately 85%) in both injury groups reported mild symptoms only (χ^2^_Yates_[1] = 0.04, *p* = 0.840). Participants with TBI scored higher than the orthopedic group on most items (*p* < 0.05), except on symptoms of “sleep disturbance” (item 5) and “restlessness” (item 16).

**Table 3.** Item-level Rasch Model Fit Statistics for the Initial Analysis of the 16-item RPQ with Item Locations, Fit Residuals, Chi-Square Statistics, and % of Participants Endorsing Symptoms by Response Category and Injury Group

							% Endorsing symptom category										
		Location	SE	Fit Resid	χ^2^	Prob	TBI					Orthopedic					Sig^a^
	RPQ item						0	1	2	3	4	0	1	2	3	4	
1	Headaches	-0.17	0.10	0.08	7.54	0.581	37	47	9	4	4	72	20	4	4	0	0.000^*^
2	Feelings of dizziness	0.65	0.12	-0.31	5.86	0.754	36	50	11	3	0	76	21	1	2	0	0.000^*^
3	Nausea and/or vomiting	1.54	0.15	1.03	5.49	0.790	56	42	2	0	0	76	23	1	0	0	0.009^*^
4	Noise sensitivity, easily upset by loud noise	0.16	0.10	-1.68	8.54	0.480	48	34	10	6	2	86	10	3	2	0	0.000^*^
**5**	**Sleep disturbance**	**-0.44**	**0.08**	**2.86**	**27.43**	**0.001**	50	24	6	16	5	46	40	5	8	0	0.571
6	Fatigue, tiring more easily	-0.87	0.09	-0.26	15.23	0.085	21	40	14	22	3	40	38	11	8	3	0.024^*^
7	Being irritable, easily angered	-0.52	0.09	-0.80	3.28	0.952	37	36	8	14	6	59	28	9	3	2	0.003^*^
8	Feeling depressed or tearful	-0.13	0.09	-0.40	5.24	0.813	45	32	12	9	2	64	27	4	4	1	0.006^*^
9	Feeling frustrated or impatient	-0.80	0.09	-1.33	8.72	0.464	22	42	16	14	6	40	46	8	4	1	0.001^*^
10	Forgetfulness, poor memory	-0.53	0.09	-0.77	7.33	0.602	19	36	21	18	6	76	12	6	5	0	0.000^*^
**11**	**Poor concentration**	**-0.30**	**0.09**	**-2.88**	**14.85**	**0.095**	38	32	12	13	6	75	17	4	4	0	0.000^*^
**12**	**Taking longer to think**	**-0.52**	**0.08**	**-2.80**	**11.34**	**0.253**	28	31	18	16	6	72	19	4	4	1	0.000^*^
13	Blurred vision	0.56	0.11	0.41	13.43	0.144	71	19	5	5	1	85	11	3	1	0	0.032^*^
14	Light sensitivity, easily upset by bright light	0.79	0.11	-0.32	8.15	0.519	61	29	6	5	0	84	10	3	4	0	0.001^*^
15	Double vision	0.91	0.18	-0.86	5.08	0.828	81	17	0	1	1	96	4	0	0	0	0.002^*^
16	Restlessness	-0.32	0.09	0.50	21.49	0.011	48	30	12	6	5	60	32	6	2	1	0.091

0 = not experienced at all; 1 = no more of a problem; 2 = mild problem; 3 = moderate problem; 4 = severe problem.

^*^ Denotes statistical significance at *p* < 0.05.

^a^ Mann-Whitney U test for comparing mean rank ordinal scores for TBI and orthopedic groups at individual item-level.

Bold indicates significant misfit to the Rasch model.

RPQ, Rivermead Post-Concussion Symptoms Questionnaire; SE, standard error; TBI, traumatic brain injury.

Summary statistics for the overall Rasch model fit are presented in Table 4. Preliminary Rasch analyses of the 16-item scale demonstrated good reliability (Person Separation Index [PSI] = 0.84) and unidimensionality; however, the data did not meet the expectations of the Rasch model due to significant interaction between items and the overall symptoms. Closer examination of the individual items presented in Table 3 showed that items “sleep disturbance,” “poor concentration,” and “longer to think” had significant misfit to the Rasch model exceeding the ±2.50 acceptable threshold. In general, items on physical symptoms such as dizziness, nausea, and vision had the highest item locations indicating that participants were less likely to endorse these symptoms than others. Affective or cognitive symptoms (e.g., sleep, fatigue frustration, and memory problems) in comparison, were more readily endorsed by participants.

**Table 4.** Summary of Fit Statistics for Initial and Final Rasch Analyses of the 16-Item RPQ

RPQ analyses	Item fit residual		Person fit residual		Goodness of fit		PSI	Significant* t *tests	
	Value / SD		Value / SD		χ^2^ (df)	P		%	% Lower bound (unidimensionality)
Initial (16)	-0.47	1.40	-0.30	0.95	111.37 (80)	0.01	0.84	9.87	0.48 (NO)
Super-items (8)^a^	-0.49	0.67	-0.37	1.02	63.77 (72)	0.74	0.87	3.59	0.73 (YES)

^a^ Super-items were created based on residual correlations: “headaches-frustrated,” “dizziness-irritability,” “nausea-longer to think,” “noise sensitivity-restlessness,” “sleep disturbance-forgetfulness,” “fatigue-poor concentration,” “depressed-blurred vision,” and “light sensitivity-double vision.”

df, degrees of freedom; PSI, Person Separation Index; RPQ, Rivermead Post-Concussion Symptoms Questionnaire; SD, standard deviation.

There were indications of DIF by injury group for items “sleep disturbance” and “forgetfulness,” which were confirmed to be artificial. DIF by age, sex, ethnic group, education level, marital status, or time since injury was not evident. Examination of the residual correlations also indicated the presence of local response dependency among several items, suggesting that responses to items may be influenced by one another due to potential method effects. To reduce measurement error affecting fit to the Rasch model and to correct for local response dependency, related items were subsequently paired based on residual correlations to create eight super-items: 1 + 9, 2 + 7, 3 + 12, 4 + 16, 5 + 10, 6 + 11, 8 + 13, 14 + 15. Analysis of the eight super-items showed a considerable improvement and yielded strong fit to the Rasch model (χ^2^[72] = 63.77, *p* = 0.74), with evidence of unidimensionality and improved reliability (PSI = 0.87).

**FIG. 1.**
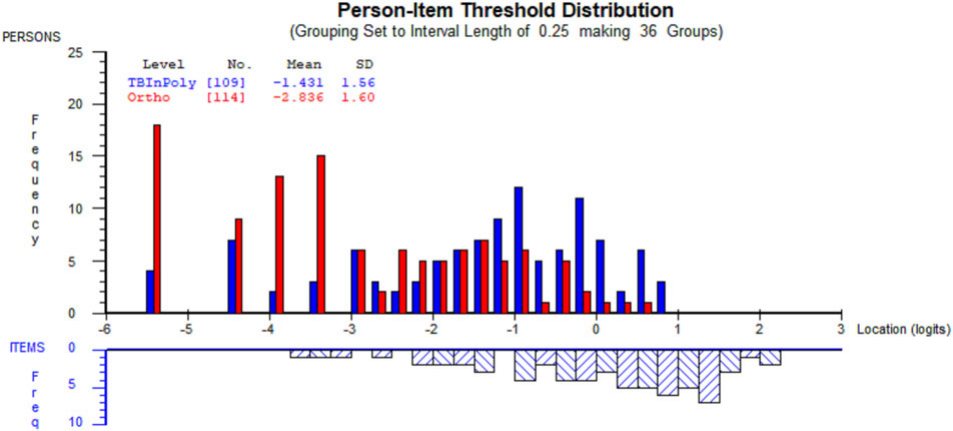
Person-item threshold distribution for the 16-item RPQ. The blue bars indicate the TBI subsample (*n* = 109) and the red bars the orthopedic subsample (*n* = 114). In the top half, negative values (locations) represent individuals on the lower end of the PCS symptoms spectrum, whereas in the bottom half, negative locations indicate items that were most frequently endorsed by the sample. Conversely, positive locations indicate those with higher levels of PCS symptoms, or items that were least readily endorsed by respondents. PCS, post-concussion syndrome; RPQ, Rivermead Post-Concussion Symptoms Questionnaire; TBI, traumatic brain injury.

As shown in Figure 1, the targeting of items to persons was suboptimal. For participants with TBI, the person mean was −1.43 (standard deviation [SD] = 1.56), deviating from the ideal value of 0.00. However, only a relatively minor floor effect was detected, with around 12% of participants with TBI not covered by the scale. In comparison, item-person targeting was poor for participants in the orthopedic sample (−2.94, SD = 1.59), who presented with no or very minor symptoms, and a thus considerable degree of floor effect (about 50%) was noticeable for this group.

Altering of scale parameters by virtue of Rasch transformation from ordinal-to-interval-level scores demonstrated a significant within-participant difference between scores (*t*[222] = −33.13, *p* < 0.001), as shown in Table 5. This means that, as per our conversions in Supplementary Table S1, a raw total score of 2 means an individual's total score is 10.40 at the interval level, having adjusted for item difficulty and person ability. As expected, participants with TBI showed higher total RPQ scores for both ordinal and Rasch-converted interval measures. Among demographic variables, younger participants appeared to report higher levels of symptoms in both TBI and orthopedic groups (Table 6). Interestingly, longer hospitalization (*r*_s_ = 0.40) and increased ISS (*r*_s_ = 0.39) correlated significantly with higher RPQ interval scores, but only among orthopedic participants (*p* < 0.001). GCS scores were not correlated with RPQ scores (*p* > 0.05) for participants with TBI.

**Table 5.** Comparisons of RPQ Rasch-Transformed-Interval and Ordinal-Level Total Scores

RPQ	Injury group	Mean	SD	Sig.
Interval scores	TBI	26.45	10.77	0.000^*,a^
	Orthopedic	16.71	11.02	
Ordinal scores	TBI	15.16	10.58	0.000^*,b^
	Orthopedic	6.94	7.36	

^*^ Significant at *p* < 0.05.

^a^ *t* test; ^b^Mann-Whitney U test.

Ordinal total scores were only calculated for the purposes of demonstrating differences with interval scores

RPQ, Rivermead Post-Concussion Symptoms Questionnaire; SD, standard deviation; TBI, traumatic brain injury.

**Table 6.** Spearman Correlations between RPQ Interval Scores, Injury Factors, Comorbidity and Quality of Life for TBI (*n* = 109), and Orthopedic (*n* = 114) Participants

Variables	RPQ interval scores	
	TBI	Orthopedic
Age	-0.22^*^	-0.30^**^
Time since injury (years)	0.06	-0.13
Length of hospital stay	0.15	0.40^**^
ISS	-0.07	0.39^**^
GCS^a^	-0.17	-0.30
Pre-injury total comorbidity^b^	0.06	0.06
Prior musculoskeletal^b^	0.05	0.17
Prior neurological^b^	0.20^*^	0.15
Prior psychological^b^	0.25^*^	0.18^*^
Post-injury total comorbidity^b^	0.27^**^	0.28^**^
Post-injury musculoskeletal^b^	0.29^**^	0.24^**^
Post-injury neurological^b^	0.33^**^	0.34^**^
Post-injury psychological^b^	0.40^**^	0.39^**^
WHOQoL-BREF total interval score^c^	-0.68^**^	-0.47^**^
Physical domain^c^	-0.58^**^	-0.47^**^
Psychological domain^c^	-0.67^**^	-0.39^**^
Social domain^c^	-0.32^**^	-0.29^**^
Environmental domain^c^	-0.65^**^	-0.40^**^

^*^ Significance at *p* < 0.05; ^**^significance at *p* < 0.001.

^a^ GCS scores, which were only available for 69% of TBI and 17% of orthopedic participants; ^b^ordinal-level comorbidity scores on the Cumulative Illness Rating Scale; ^c^total interval and domain-level scores for the 24-item WHOQoL-BREF excluding global items 1 and 2.

GCS, Glasgow Coma Scale; ISS, Injury Severity Score; RPQ, Rivermead Post-Concussion Symptoms Questionnaire; WHOQoL-BREF, World Health Organization Quality of Life Brief Version.

Although pre-injury total comorbid illness as measured by the CIRS did not have an association with total RPQ scores, prior neurological and psychological problems did show a weak association with PCS symptoms (*r*_s_ = 0.20 − 0.25), especially for those with TBI. Post-injury comorbidity in comparison, demonstrated stronger associations with RPQ scores in both groups (*r*_s_ = 0.27 − 0.28), particularly for post-injury neurological (*r*_s_ = 0.33 − 0.34) and psychological disturbances (*r*_s_ = 0.39 − 0.40). RPQ interval scores demonstrated good concurrent validity with WHOQOL scores in both samples (*r*_s_ = −0.47 to −0.68), notably for the physical (*r*_s_ = −0.47 to −0.58), psychological (*r*_s_ = −0.39 to −0.67), and environmental domains (*r*_s_ = −0.40 to −0.65).

## Discussion

The aim of this Rasch examination of the RPQ was to explore the dimensionality of persistent PCS symptoms occurring at 2.5 years after TBI, and to clarify previous inconsistencies on factor structure. Further, the study aimed to validate the RPQ for use with non-TBI samples, which has not been done previously. Our findings indicate that the RPQ achieves good fit to the Rasch model, demonstrating strong reliability in both TBI and orthopedic groups. A reliability coefficient of 0.87 is congruent with values reported by other Rasch studies,^11,12^ and indicates the measure's usefulness across group comparisons, as well as for individual assessment of symptoms. Further examination indicates that the scale covers close to 90% of the TBI sample, and items function invariantly by age, sex, marital status, ethnicity, time since injury, and injury type. RPQ interval total scores had strong concurrent validity with quality-of-life outcomes and were correlated with post-injury neurological and psychological comorbid illness.

Utilizing the super-item approach and thereby attenuating the presence of method effects, we presented clear evidence on the structural validity of the RPQ, as representing a unidimensional construct of PCS symptoms for both TBI and orthopedic patients. Multi-dimensionality as reported previously may not be due to *local trait dependency*, but instead to responses across items influencing each other (*local response dependency*), or method effects, as shown by our study.

Our results are in contrast with other studies using both Rasch and factor analysis that have so far lent support to multi-dimensionality, comprising various factor solutions.^11,12,29–31^ A previous Rasch study by Eyres and colleagues^11^ found evidence of a two-dimensional model consisting of a mixture of somatic and psychosomatic symptoms for patients with TBI assessed between 3 and 6 months post-injury. Lannsjö and colleagues^12^ in their Rasch analysis employed a combination of item rescoring and the super-item approach, but were not able to resolve underlying issues around significant item-trait interaction noted in the scale. They concluded the possibility of three or more dimensions underpinning PCS symptoms for patients at 3 months post-TBI. Inconsistent results and persistent ambiguity around factor structure of the RPQ has to date led to challenges in the calculation and interpretability of summary scores.^14^ The evidence presented in this study therefore alleviates some of this uncertainty about factor structure of PCS symptoms.

It is worth noting that in the two Rasch studies previously conducted on the RPQ, and other investigations using factor analysis, symptoms were usually assessed in the acute symptom stage, typically within the first 3 to 6 months after TBI. These studies are marked by fluctuating symptom presentation in patients, which may be reflected in some of the collective evidence for a multi-dimensional structure of symptoms appearing in the first year after TBI. In contrast, in our study symptoms were assessed within a mid-late recovery period, at approximately mean 2.5 years after injuries, which may partly explain our findings for a unidimensional construct of PCS symptoms in both groups. One may postulate whether this finding of unidimensionality may be attributed to the existence of long-term stable symptoms that occur after the experience of an injury. Evidence from a study by Barker-Collo and colleagues^31^ seem to support the notion that temporal changes occur within the factor structure for the TBI population, in which symptom structure is best defined by three factors in the first month, whereas two factors appear to describe the data better at 6 and 12 months. The authors thus concluded that there appears to be a relative stability in factor structure after 6 months, distinguishing between dynamic or early symptoms that are present in the first 3 months, and the persistence of more stable symptoms thereafter.

A later investigation by Barker-Collo and colleagues^13^ revealed evidence for a one-factor structure of PCS symptoms appearing among patients with TBI at 4 years post-injury. This concept of transient versus stable dimensions of PCS symptoms was further explored statistically by Medvedev et al.^32^ using *Generalizability Theory*. It was concluded from their analysis that although the RPQ is a reliable measure in assessing enduring symptoms at 6–12 months, it is however limited in its ability to assess dynamic symptoms that fluctuate across the initial days and weeks following TBI. Few studies exist on the temporality of PCS symptoms; however, the available evidence does collectively seem to suggest that symptom dimensions are likely to amalgamate as the time elapsed since injury increases.

Detailed examination of the individual items in our analysis confirmed previous Rasch work that items function invariantly by age and sex.^11,12^ Another strength of our study is that we were able to assess DIF by injury group and showed that RPQ items function effectively among those with TBI and orthopedic injuries, for ethnicity, and for symptoms in the early to mid-recovery and mid to late-recovery periods. These findings indicate that the unitary construct of the RPQ is similar for both TBI and orthopedic populations, and that the RPQ can be reliably administered to both populations as a measure of long-term symptoms. It remains to be seen whether the structure of symptoms among orthopedic patients will follow a trajectory similar to that in patients with TBI—from symptoms appearing multi-dimensional in the first few months after injury, to a more unidimensional structure with increased time.

In the extant literature, the evidence gathered so far on PCS symptoms appears to fuel the long-standing debate as to whether the appearance of this constellation of symptoms is unique to individuals with TBI. The lack of specificity of the RPQ is further empirically supported by evidence from studies on the existence of PCS-like symptoms in various groups such as among patients with chronic pain,^15^ depression,^33^ orthopedic injuries,^17^ and even in healthy samples.^8,34^ As early as the 1980s, several researchers had already begun to question the specificity of this phenomenon.^35–37^ In fact, Lishman^35^ argued that there is no apparent demarcation between symptoms that are solely attributable to neurobiological mechanisms of a cerebral injury, known as *physiogenic factors*, and symptoms that occur circumstantially to the experience of an injury, traumatic experience, or those masked as daily stressors. The latter have been collectively termed *psychogenic factors*. It is also important to note that these factors do not need to occur in a mutually exclusive manner, but can arise in combination.^38^ Aside from these theoretical debates on the misclassification of PCS, the evidence nevertheless points to the commonality in symptoms with non-TBI individuals. This evidence needs to be explored further by methods such as network analysis,^39^ and should include further validation of PCS symptom scales for their potential use in other populations, which is currently lacking.

The achievement of a unidimensional fit of the RPQ discussed earlier has several implications, the first being that it is a prerequisite for the calculation of a single total score.^40^ The conversion to an interval measurement provided herein allows the summation of item scores, which can facilitate clinicians' assessment of change scores at various stages of a patient's recovery. From a statistical perspective, these conversions from ordinal to interval level also strengthen the precision of the scale by permitting the use of parametric testing methods that would otherwise violate the required assumptions. Our comparisons between the ordinal scores and interval scores showed a considerable difference, where corresponding mean interval scores on the RPQ would result in an increase by a mean of 10 points. The conversion table shows that the magnitude of score differences is mostly concentrated in the lower-most end of the spectrum, among individuals who are seemingly asymptomatic but who may actually be experiencing at least some symptoms of PCS. Last, having demonstrated unidimensionality and conversion to interval total scores, this study may serve as a starting point for researchers to develop thresholds for the RPQ to be used as a diagnostic instrument. This may help to determine to what degree collective symptoms indicate the severity of PCS in an individual, and at what cutoff score clinical intervention may be deemed necessary.

### Limitations

Some study limitations need to be considered. First, this study represents a cross-sectional snapshot of symptoms reported at different time intervals, with a mean time centering around 2.5 years post-injury. Therefore, for some participants, especially those in the later stages of recovery, their experience of symptoms may not necessarily indicate late sequelae of injuries, but rather environmental stressors, for example, life events or stress-coping mechanisms.^41^ Also, in our study we found that the coverage was poor for the orthopedic sample, with only 65% coverage by items. Although this may not be seen as an outright limitation, it suggests that the informational value provided by the RPQ for the orthopedic sample is limited, especially for those presenting with no to very minor symptoms. As ours is the only study to have attempted validation of a PCS measure in this population, further research in this population is needed to substantiate these findings.

Another limitation is that the assumption that most of the TBI cases were mild was based on GCS scores, which were only available for two-thirds of the sample. Lack of complete data or inconsistent recording of GCS scores are not unique to our study; they are common occurrences in hospital registries.^42^ In addition, as case patients were recruited from hospitalizations, it may be that our sample represents patients with more “complicated” injuries within the spectrum, who may differ in symptom presentation compared with those with “uncomplicated” mild cases who do not present to the hospital.^43^ It is also likely that some individuals with orthopedic injuries may not have been assessed for the possibility of a TBI, thereby undercounting the true extent of individuals with TBI in our sample. In our study, isolated TBI cases were combined with multiply injured TBI cases (i.e., TBI with extracranial injuries) to improve statistical power for analysis, which may have inadvertently introduced some degree of confounding.

Last, we acknowledge that different approaches are undertaken to derive total scores, with King and colleagues^9^ excluding all responses of 1, “no more of a problem,” to detect change in symptoms since injury. Other studies including for Rasch analysis have either combined response categories 0, and 1,^11^ or included all response categories in the total score.^3,12^ Our analysis followed the convention used by Lannsjö and associates^12^ to include all scores in the calculation of the total score, given that the assumption of unidimensionality has been met. In the extant literature there exists no gold standard on how the RPQ should be scored, and therefore care needs to be taken during interpretation of total scores, particularly when comparing between individuals.

## Conclusion

Our findings through Rasch analysis confirm that the RPQ remains a reliable measure that can be used as a tool for individual assessment of long-term PCS symptoms in both TBI and orthopedic populations. Utilizing a subtest approach to correct for the presence of method effects in items, our results produced evidence of a unidimensional construct of symptoms, which helps elucidate previous inconsistent findings on its structural validity. The conversion of scores provided allow for the calculation of summary scores and is a useful starting point for comparing between individuals for clinical and research purposes, and possibly for the future development of cutoff total scores to establish clinical thresholds. Further research is needed to ascertain whether the RPQ can be used as a reliable measure in other populations that have been found to experience PCS-like symptoms.

## Supplementary Material

Supplemental data

## Abbreviations Used

CIRS: Cumulative Illness Rating Scale
df: degrees of freedom
DIF: differential item functioning
GCS: Glasgow Coma Scale
ISS: Injury Severity Score
LOS: LOS, length of hospital stay in days
NZ: New Zealand
PCS: post-concussion syndrome
PSI: Person Separation Index
RPQ: Rivermead Post-Concussion Symptoms Questionnaire
RUMM: Rasch Unidimensional Measurement Model
SD: standard deviation
SE: standard error
TBI: traumatic brain injury
WHOQoL-BREF: World Health Organization Quality of Life Brief Version
