# Supplementary material for: Is the Rivermead Post-Concussion Symptoms Questionnaire a Reliable and Valid Measure to Assess Long-Term Symptoms in Traumatic Brain Injury and Orthopedic Injury Patients? A Novel Investigation Using Rasch Analysis
Source: Neurotrauma Rep. 2020 Aug 11;1(1):63–72. doi: 10.1089/neur.2020.0017 (PMC8240882; doi:10.1089/neur.2020.0017)
Supplement: Supplemental data [file Supp_TableS1.pdf]

Supplementary Data

Supplementary Table S1. Ordinal to Interval Conversions for Total RPQ Scores

Ordinal Total score	Interval Logits	Interval Scale	Ordinal Total score	Interval Logits	Interval Scale
0	-5.35	0.00	33	0.37	39.68
1	-4.48	6.08	34	0.43	40.11
2	-3.86	10.40	35	0.49	40.53
3	-3.41	13.46	36	0.55	40.95
4	-3.07	15.88	37	0.61	41.36
5	-2.77	17.90	38	0.67	41.77
6	-2.52	19.65	39	0.72	42.16
7	-2.30	21.19	40	0.78	42.56
8	-2.10	22.57	41	0.84	42.95
9	-1.92	23.82	42	0.89	43.33
10	-1.75	24.97	43	0.95	43.71
11	-1.60	26.04	44	1.00	44.08
12	-1.46	27.03	45	1.06	44.46
13	-1.33	27.95	46	1.11	44.83
14	-1.20	28.81	47	1.16	45.19
15	-1.08	29.62	48	1.21	45.55
16	-0.97	30.38	49	1.27	45.92
17	-0.87	31.11	50	1.32	46.30
18	-0.77	31.80	51	1.38	46.69
19	-0.68	32.45	52	1.43	47.07
20	-0.59	33.08	53	1.49	47.49
21	-0.50	33.69	54	1.56	47.93
22	-0.42	34.26	55	1.62	48.41
23	-0.33	34.82	56	1.70	48.93
24	-0.26	35.36	57	1.79	49.52
25	-0.18	35.89	58	1.88	50.21
26	-0.11	36.40	59	2.00	51.03
27	-0.03	36.91	60	2.15	52.05
28	0.04	37.40	61	2.35	53.41
29	0.11	37.87	62	2.63	55.38
30	0.17	38.34	63	3.10	58.64
31	0.24	38.79	64	3.87	64.00
32	0.30	39.24			

RPQ, Rivermead Post-Concussion Symptoms Questionnaire.
